# Cardiovascular Pleiotropic Effects of Natriuretic Peptides

**DOI:** 10.3390/ijms20163874

**Published:** 2019-08-08

**Authors:** Maurizio Forte, Michele Madonna, Sonia Schiavon, Valentina Valenti, Francesco Versaci, Giuseppe Biondi Zoccai, Giacomo Frati, Sebastiano Sciarretta

**Affiliations:** 1 IRCCS NEUROMED, 86077 Pozzilli, Italy; 2 Department of Medico-Surgical Sciences and Biotechnologies, Sapienza University of Rome, 04100 Latina, Italy; 3 Department of Cardiology, Santa Maria Goretti Hospital, 04100 Latina, Italy; 4 Mediterranea Cardiocentro, 80122 Napoli, Italy

**Keywords:** Atrial Natriuretic peptide, natriuretic peptides, cardiac remodelling, cardiac hypertrophy, vascular homeostasis

## Abstract

Atrial natriuretic peptide (ANP) is a cardiac hormone belonging to the family of natriuretic peptides (NPs). ANP exerts diuretic, natriuretic, and vasodilatory effects that contribute to maintain water–salt balance and regulate blood pressure. Besides these systemic properties, ANP displays important pleiotropic effects in the heart and in the vascular system that are independent of blood pressure regulation. These functions occur through autocrine and paracrine mechanisms. Previous works examining the cardiac phenotype of loss-of-function mouse models of ANP signaling showed that both mice with gene deletion of ANP or its receptor natriuretic peptide receptor A (NPR-A) developed cardiac hypertrophy and dysfunction in response to pressure overload and chronic ischemic remodeling. Conversely, ANP administration has been shown to improve cardiac function in response to remodeling and reduces ischemia-reperfusion (I/R) injury. ANP also acts as a pro-angiogenetic, anti-inflammatory, and anti-atherosclerotic factor in the vascular system. Pleiotropic effects regarding brain natriuretic peptide (BNP) and C-type natriuretic peptide (CNP) were also reported. In this review, we discuss the current evidence underlying the pleiotropic effects of NPs, underlying their importance in cardiovascular homeostasis.

## 1. Introduction

Atrial natriuretic peptide (ANP) was the first member of the natriuretic peptides (NPs) family to be discovered, in 1981 [1,2,3]. The other two members, brain natriuretic peptide (BNP) and C-type natriuretic peptide (CNP) were identified a few years later [4,5]. NPs share some similarities: All three peptides are encoded by genes including three exons and display a 17-amino-acid ring structure in their active forms [6,7]. NPs are synthetized as pre-hormones and subsequently cleaved into the biological active carboxy terminal forms (α-ANP, BNP-32, CNP-22), together with their respective amino-terminal ends. The latter are the more stable circulating form of NPs [6,7,8]. ANP and BNP are synthetized prevalently in the heart, atria, and ventricles, respectively. However, a lesser expression of ANP and BNP has been reported in other areas [6,7,8]. Endothelium is the principal source of CNP production [6,7,8].

The systemic effects of NPs are well described in the literature [9,10]. They are prevalently secreted in response to the mechanical stretch of myocardial walls induced by volume or pressure overload and then, once secreted, they exert diuretic, natriuretic, and vasodilatory effects, thereby maintaining cardio–renal homeostasis and hemodynamic status through the regulation of water–salt balance and body fluid volume [9,10,11]. NP secretion is also induced by other hormones, such as endothelin 1, angiotensin II, and by the adrenergic system [12,13,14]. In turn, NPs inhibit the renin–angiotensin–aldosterone system (RAAS) and sympathetic nervous system (SNS) [3,15]. Based on all these effects, NPs may be considered as pivotal players in the pathophysiology of hypertension. Mice with homozygous genetic ablation of the ANP gene display absent levels of circulating ANP and develop salt-sensitive hypertension [16]. For these reasons, several therapeutic strategies for the management of hypertensive subjects have been created with the aim of increasing circulating levels of NPs [9,10]. NPs are also valid diagnostic and prognostic markers in cardiovascular diseases (CVDs), such as heart failure (HF), coronary artery diseases, valvular diseases, myocardial infarction, and stroke, also in apparently healthy individuals [17,18,19,20,21].

Among the NPs, both the active (BNP) and the inactive forms (either glycosylated or not-glycosylated proBNP and NTpro-BNP) of BNP are considered as the first line biomarkers for the diagnosis and progression of acute and chronic HF [22]. This may be due to the longer half-life and stability of BNP as compared to the other NPs (see below). Circulating levels of BNP and NTproBNP have been shown to directly correlate with the clinical outcomes in patients with HF and to be more accurate if compared to other markers used routinely, such as troponin [22]. However, the three forms of BNP detected in plasma shared different analytical properties, and to date there are no valid commercial kits able to detect only the active form of BNP, which is the form with the shorter half-life. The latter is in part attributable to the cross-reactivity of antibodies with glycosylated and not-glycosylated forms of proBNP. Of note, the inactive forms of BNP display a lower intra-individual biological variation and they have a higher ability to predict HF progression as compared to BNP. For these reasons, it is recommended to consider the level of BNP and proBNPs together in HF patients [22]. It is also strongly recommended that NPs levels should be evaluated in conjunction with other clinical parameters, such as renal function, body mass index, and cardiac imaging [23].

Besides their systemic effects, accumulating lines of evidence indicate that ANP has beneficial pleiotropic effects on the cardiovascular system at baseline and in response to stress. These pleiotropic functions are mediated by paracrine and autocrine mechanisms and are independent of blood pressure regulation [9,24]. ANP was shown to reduce maladaptive cardiac remodeling, exerting anti-hypertrophic and anti-fibrotic effects in response to pressure overload and chronic ischemic remodeling [25,26,27,28]. ANP administration reduces ischemia-reperfusion (I/R) injury. In addition, it was previously demonstrated that ANP induces anti-inflammatory and pro-angiogenetic effects in the vascular system [29,30,31]. On the other hand, similar effects do not seem to be induced by BNP, which is only able to exert anti-fibrotic functions [9,32].

In this review, we will discuss the pleiotropic beneficial effects of natriuretic peptides in the heart and vascular system, with a particular focus on ANP. We will summarize the results obtained in loss-of-function mouse models of ANP signaling undergoing cardiac and metabolic stress. We also discuss the available human evidence regarding the pleiotropic effects of ANP in the cardiovascular system.

## 2. Overview of ANP Metabolism

Once synthetized, the inactive form of ANP (pro-ANP) is stored in secretory granules of atrial cardiomyocytes [8]. Following this, pro-ANP is cleaved by the atrial natriuretic peptide-converting enzyme (Corin) in α-ANP and NT-proANP, which are the two detectable plasmatic forms [10]. Accordingly, corin knockout (KO) mice showed reduced level of active ANP and develop a mild hypertension [33]. The proprotein convertase subtilisin/kexin type 6 (PCSK6) is another important member of NP metabolic cascade. PCSK6 cleaves and activates the zymogen corin. PCSK6 KO mice develop salt-sensitive hypertension, with the consequent inhibition of ANP processing and corin activity in the heart. Notably, a genetic variant in the catalytic domain of PCSK6 was found in hypertensive patients. When transfected in cells, this variant of PCSK6 was unable to activate corin [34]. Similarly, genetic variants in corin gene that associate with hypertension and heart diseases were reported to impair PCSK6-mediated corin activation [35,36,37].

ANP exerts its biological effects by the interaction with the natriuretic peptide receptor type A (NPR-A). BNP also interacts with NPR-A, whereas CNP binds with a higher affinity to the type B receptor (NPR-B) [38]. NPR-A and NPR-B are widely distributed in the body. They are prevalently expressed in the kidney, brain, vascular system, heart, adrenal gland, and pancreas [39,40]. The adipose tissue is also a target of NPs. In this regard, NPRs are highly expressed in human adipose cells and both ANP and BNP were found to regulate lipid metabolism, by promoting lipolysis and adiponectin secretion [41,42,43,44,45]. This evidence suggests an important cross-talk between cardiac and adipose tissue [46] Both NPR-A and B are coupled to a guanylate cyclase (GC) and induce an increase of intracellular levels of cyclic guanosine monophosphate (cGMP) upon their activation [9,10,47]. Specifically, NPR-A and NPR-B are characterized by an external region, devoted to the interaction with NPs, a membrane region and an intracellular region containing the GC catalytic domain [48]. An additional receptor, NPR-C, does not show GC activity and it is instead coupled with an inhibitory G protein (G_i_α), which leads to the reduction of intracellular cyclic adenosine monophosphate (cAMP) levels, when NPR-C is activated. NPR-C contains an external region, which is the homologous of the external region of NPR-A and NPR-B. In contrast, its intracellular region is composed by only 37 amino acids. NPR-C is responsible for the clearance of NPs [47,49]. The expression of NPR-C has been reported in heart, vascular cells, pancreas, gastrointestinal tract, neurons, and chondrocytes [40]. The clearance of NPs mediated by NPR-C occurs through its internalization and delivery to lysosomes. However, known cytoplasmic international motifs have not been identified in NPR-C sequences [48]. It has been reported that the removal of ANP from circulation occurs mainly in the lung, liver, and kidney [50]. Clearance of ANP ranges from 0.5 min to 4 min [48], which is comparable with the clearance of other vasoactive hormones, such as angiotensin II and vasopressin [51,52]. BNP displays the longest half-life (about 23 min) among NPs, whereas CNP has the shortest half-life, which was very close to ANP (about 3 min) [53,54,55]. The differences between the half-life of NPs are attributable to their different binding affinity with NPR-C. In this case, ANP displays the major affinity with NPR-C. However, aside from its functions as a clearance receptor, it is now well established that NPR-C also mediates important cellular functions of NPs, especially CNP.

Besides NPR-C, the degradation of circulating NPs is also achieved by the neutral endopeptidase neprilysin (NEP) [56,57,58]. NEP is ubiquitously expressed in the body. It also cleaves other vasoactive peptides, such as angiotensin I and II, bradichinin [59]. The NPs degradation mediated by NEP occurs when NPR-C is saturated [60]. Recently, drugs combining NEP inhibition with angiotensin II receptor inhibition, named ARNi, have been introduced in the management of patients with HF, in order to maintain high levels of circulating NPs, as discussed in detail below [61,62].

## 3. ANP and Cardiac Pleiotropic Effects

**Figure 1:**
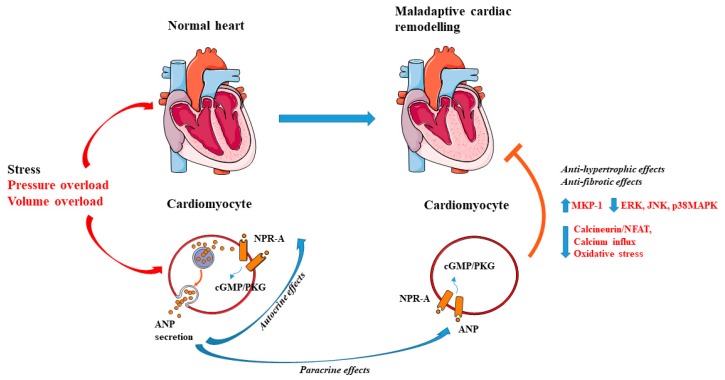
Local action of atrial natriuretic peptide (ANP) in cardiomyocytes. ANP is secreted by cardiomyocytes in response to cardiac stress. After secretion, ANP binds to the natriuretic peptide receptor (NPR-A) and activates the cGMP/PKG pathway. Anti-hypertrophic and anti-fibrotic effects of ANP occur through autocrine and paracrine mechanisms. Arrow-headed lines indicate activation, whereas bar-headed lines indicate inhibition. Legend: c-Jun N-terminal kinase (JNK); cyclic guanosine monophosphate (cGMP); extracellular signal-regulated kinase (ERK); mitogen-activated protein kinase phosphatase-1 (MKP-1); natriuretic peptide receptor type A (NPR-A); nuclear factor of activated T-cells (NFAT); p38 mitogen-activated protein kinase (MAPKs); protein kinases G (PKG). See text for further details. The figure was made using tools provided by Servier Medical Arts, amongst others.

The expression of NPR-A in cardiac cells, both in myocytes and in fibroblasts suggests a fundamental role played by ANP in the heart through autocrine and paracrine mechanisms (Figure 1) [63,64]. In fact, in vitro studies revealed that ANP inhibits cell growth and proliferation in cardiomyocytes and promotes apoptosis [65,66,67]. The observation underlying the anti-hypertrophic effects of ANP has been extensively investigated in KO models of ANP and NPR-A. For example, it was shown that mice with genetic disruption of ANP develop cardiac hypertrophy and hypertension in response to chronic hypoxia (3 to 5 weeks) and high salt diet administration [16,68,69,70]. Feng et al. demonstrated that the observed cardiac hypertrophy found in ANP KO models was independent of change in blood pressure. In fact, when the blood pressure was normalized by a low salt diet regimen ANP KO mice also developed cardiac hypertrophy, compared to wild-type animals [71]. In addition, ANP deficient mice undergoing 2 weeks of volume overload through an aorto-caval fistula showed left ventricular hypertrophy (LVH) and dysfunction when fed with both a regular and a low salt diet [27]. Franco et al. [24] further investigated the development of cardiac hypertrophy in heterozygous ANP KO mouse after 1 week of pressure overload induced by transverse aortic constriction (TAC). The authors showed that the partial inhibition of ANP also leads to hypertrophy and adverse cardiac remodeling either at baseline and after hemodynamic stress without any significant effect on blood pressure. Conversely, the exogenous administration of ANP was found to be sufficient to reduce cardiac remodeling in a model of chronic myocardial infarction [72]. Kinoshita et al. demonstrated that ANP exerts its anti-hypertrophic actions on cardiac myocytes through the inhibition of the transient receptor potential subfamily C (TRPC-6), in a protein kinases G (PKG) mediated manner. TRPC-6 triggers hypertrophic stimuli by activating the calcineurin–nuclear factor of activated T cells (NFAT) signaling. In the same study, the authors found that inhibition of PKG, as well as modifications in the phosphorylation site of TRPC-6, blunts the anti-hypertrophic actions of ANP [73].

Interesting findings have also been obtained in KO of NPR-A (NPR-A KO). NPR-A null mice are characterized by cardiac hypertrophy and chamber dilatation at three months of age. These effects were not exclusively attributable to the observed increase of blood pressure levels in these animals, since the magnitude of cardiac hypertrophy was exaggerated with respect to what could be expected based on the severity of hypertension. In addition, similar levels of cardiac hypertrophy could not be observed in other mouse models of hypertension [74]. Knowles et al. corroborated these data in NPR-A KO undergoing TAC. The authors showed the exacerbation of cardiac hypertrophy and cardiac dysfunction in NPR-A null mice compared to wild-type mice; they also reported that chronic administration of different anti-hypertensive drugs, such as enalapril, furosemide, hydralazine, propranolol, and losartan was not able to reduce cardiac mass in NPR-A KO [75]. Consistently, cardiac overexpression of NPR-A reduced cardiomyocyte size in both wild-type and NPR-A KO mice, along with the reduction of ANP mRNA levels [76]. These changes were not associated with hemodynamic alteration [76]. To test the local effect of ANP in determining cardiomyocytes size, Holtwick et al. generated a model with cardiomyocyte-specific gene deletion of NPR-A (NPR-AcKO). They demonstrated a hypertrophic cardiac response in NPR-AcKO at baseline, which was accelerated in mice subjected to aortic constriction and analyzed 10 days after surgery. Moreover, in NPR-AcKO mice undergoing surgery, the authors observed a reduction of blood pressure levels, probably attributable to the increase of plasma ANP levels [26]. These findings support the concept that ANP acts as an intrinsic regulator of cardiac hypertrophy [25]. Mechanistically, it was demonstrated that ANP reduces hypertrophy induced by angiotensin-II (Ang-II) and endothelin-1 (ET-1), by increasing mitogen-activated protein kinase phosphatase-1 (MKP-1) signaling in a cGMP-dependent manner in cultured cardiomyocytes [77]. MKP-1 activation inhibits extracellular signal-regulated kinases (ERKs), c-Jun N-terminal kinases (JNK), and p38MAPKs, known inducers of cell proliferation and hypertrophy [78]. ANP was also reported to reduce protein synthesis in cardiomyocytes and fibroblasts in vitro treated with adrenergic stimuli by the inhibition of calcium influx mediated by norepinephrine [79]. The involvement of the cardiac calcineurin-NFAT pathway in cardiac hypertrophy was also reported in the NPR-A KO mice [80]. In addition, exogenous administration of ANP was able to inhibit calcineurin/NFAT signaling in cultured cardiomyocytes treated with phenylephrine [73,80]. Other studies showed that the anti-hypertrophic effects of ANP are associated with the reduction of oxidative stress in isolated cardiomyocytes treated with Ang-II and ET-1 [81]. However, most of the previous studies dissecting the mechanisms of the antihypertrophic effects of ANP were associative and further investigations are warranted to address this issue.

Human evidence also underlines the association between ANP and cardiac mass. In this regard, Rubattu et al. found that reduced levels of circulating ANP were associated with an increase in left ventricular mass in individuals with essential hypertension. Similar data were obtained in the same study in subjects carrying an allelic variant of ANP gene promoter, which is associated with reduced circulating ANP levels [82]. Similarly, a deletion mutation in the NPR-A gene was found to be associated with the development of LVH without hypertension [83]. ANP levels were found to be lower in the presence of obesity and metabolic syndrome, because of either increased clearance or reduced synthesis. [84]. Accordingly, plasma levels of ANP were found to be inversely correlated with the increase of cardiac mass in hypertensive subjects with metabolic syndrome or obesity [85,86].

## 4. ANP and Ischemia/Riperfusion Injury

Pre-clinical and clinical studies showed that ANP is able to attenuate I/R injury. ANP was found to protect isolated rat hearts from I/R injury and to increase post-ischemic cGMP level when administered at the time of reperfusion [87]. Similar findings were obtained in isolated rabbit hearts, in which infusion of ANP prior to reperfusion was reported to significantly decrease infarction area. Similarly, administration of a cell-permeable cGMP analogue was able to mimic the protection exerted by ANP. In contrast, the mitochondrial ATP sensitive potassium channel (mKATP) inhibitor 5-Hydroxyde-canoate (5-HD) blunted it [88]. Other studies revealed that the pre-ischemic infusion of ANP in isolated rat hearts was unable to limit I/R injury when the hearts were co-treated with N-nitro l-arginine methyl ester (L-NAME) or a protein kinase C (PKC) synthetase inhibitor or with a mKATP channel blocker. The latter findings suggest that the nitric oxide (NO)-PKC pathway and the mKATP channel activation are likely involved in the beneficial effects of ANP during I/R [89].

The role of ANP was also observed in models of I/R in vivo. For example, in dogs undergoing 30-min of ischemia followed by 60 min of reperfusion, ANP was shown to decrease ventricular extrasystoles and atrial fibrillation when administered either during artery occlusion or during reperfusion. An increase of myocardial ATP was found in the ischemic myocardium of ANP-treated animals. On the other hand, no differences were observed in hemodynamic parameters, suggesting that the protective effects of ANP were mediated prevalently by the elevation of cGMP [90]. In another study, ANP administration during ischemia or immediately before reperfusion was found to limit cardiac injury in pigs [91]. In pigs subjected to 30 min of ischemia followed by 4 h of reperfusion, ANP was reported to decrease myocardial injury, in association with the increase of myocardial expression of peroxisome proliferator activated receptor γ, a transcription factor involved in myocardial protection during I/R [92,93]. Of interest, Charan et al. showed that ANP was able to restore the cardioprotection conferred by ischemic pre-conditioning (four cycles of 5 min of ischemia followed by 5 min of reperfusion) in diabetic hearts, likely through an improvement of NO metabolism [94]. NPR-A KO subjected to myocardial infarction by permanent ligation of left coronary artery showed higher mortality within 1 week, as compared to wild-type mice and also a reduced water and sodium excretion. In addition, NPR-A KO mice showed exacerbated cardiac hypertrophy, fibrosis and dysfunction 4 weeks after myocardial infarction. Notably, cardiac fibrosis was absent in NPR-A mice carrying a deletion in Ang-II type 1A receptor whereas the higher mortality and cardiac hypertrophy remained unaltered [95].

Similar effects were observed in human patients with acute myocardial infarction (AMI). In fact, Hayashi et al. reported that ANP improves left ventricular ejection fraction (LVEF) and prevents left ventricular enlargement in patients with anterior AMI receiving reperfusion therapy. In the same study, the authors showed the suppression of the renin-angiotensin-aldosterone system and endothelin-1 (ET-1) pathways, known mediators of left ventricular remodeling [96]. Moreover, ANP administered immediately after coronary angioplasty limited I/R injury, reduced ST-segment elevation and increased LVEF in AMI patients [97]. Kasama et al. studied the effects of ANP on left ventricular remodeling in patients with first anterior AMI. In this study, ANP was continuously infused before and after primary coronary angioplasty. ANP drastically reduced I/R injury, inhibited LV remodeling, and ameliorated LV function, together with the reduction of cardiac sympathetic nerve activity [98]. In the J-WIND (Japan-Working Groups of Acute Myocardial Infarction for the Reduction of Necrotic Damage) clinical trial, ANP infusion was shown to decrease infarct size and to limit reperfusion injury in patients affected by AMI undergoing reperfusion therapy [99].

## 5. ANP and Vascular Pleiotropic Effects

Previous evidence indicates that ANP can be expressed and secreted by aortic endothelial cells [100], suggesting a local action of ANP also in the endothelium. Mice with endothelial-specific deletion of NPR-A gene (NPR-A-ecKO) undergoing limb ischemia displayed impaired angiogenesis until 5 weeks post-surgery. In addition, NPR-A-ecKO observed 10 days after TAC showed a decreased capillary density in the heart, which was associated with the development of cardiac hypertrophy and fibrosis. In the same study, ANP was also able to induce endothelial cell proliferation and angiogenesis in vitro [101]. Other evidence has demonstrated that physiological doses of ANP are able to induce endothelial cell proliferation and migration along with increase of phospho-Akt and phospho-ERK1/2. In contrast, excessive doses of ANP leads to opposite effects [29]. Of interest, we reported that a molecular variant of ANP (C2238-αANP), which is associated with increased cardiovascular risk, severely affects endothelial function in vitro and ex vivo in isolated mouse arteries [102,103,104,105]. C2238-αANP interacts with NPR-C receptors, leading to the inhibition of the cAMP/Akt/protein kinase A (PKA) pathway and activation of NADPH oxidase. The latter contributes to the increase of reactive oxygen species (ROS) and promotes endothelial dysfunction [102]. We also found that subjects carrying the C2238-αANP gene variant showed endothelial dysfunction [102].

**Figure 2:**
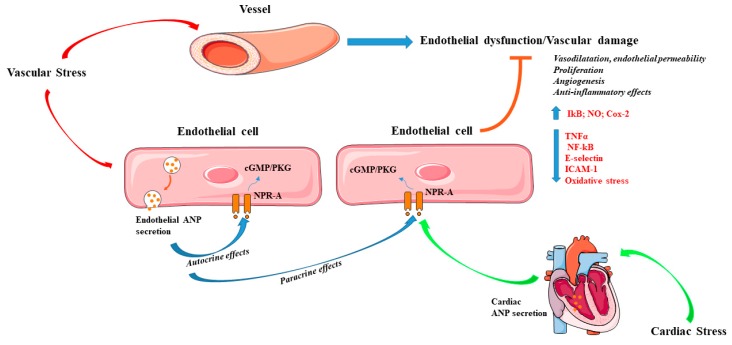
Effects of atrial natriuretic peptide (ANP) in the vascular system. The contribution of either endothelial and cardiac-derived ANP in response to stress prevents endothelial dysfunction and vascular damage. ANP increases vasodilatation and endothelial permeability, stimulates angiogenesis, proliferation, nitric oxide production, and exerts anti-inflammatory effects. Arrow-headed lines indicate activation whereas bar-headed lines indicate inhibition. Legend: cyclic guanosine monophosphate (cGMP); ciclooxygenases-2 (Cox-2); intercellular Adhesion Molecule 1 (ICAM-1); natriuretic peptide receptor type A (NPR-A); nitric oxide (NO); nuclear factor kappa-light-chain-enhancer of activated B cells (NFkB); protein kinases G (PKG); tumour necrosis factor alpha (TNFα). See text for further details. The figure was made using tools provided by Servier Medical Arts, amongst others.

Kiemer et al. reported that ANP reduces inflammation in endothelial cells by inhibiting the TNF-α-induced expression of adhesion molecules, such as E-selectin and ICAM-1. The latter is achieved by the activation of the nuclear factor of kappa light polypeptide gene enhancer in B-cells inhibitor (IkB) and the consequent inhibition of the nuclear factor kappa-light-chain-enhancer of activated B cells (NF-κB), in a cGMP-dependent manner [106]. In separate studies, the same group found that ANP is able to inhibit the macrophage expression of ciclooxygenases-2 (Cox-2) induced by lipopolysaccharides and to reduce inducible nitric oxide synthase (iNOS) expression [107,108]. Of note, inhibition of neprilysin was shown to potentiate the effects of ANP and to limit polymorphonuclear neutrophil-vascular cell interactions in vitro under hypoxia [109]. Although the evidence described above suggests that ANP may preserve vascular function through autocrine and paracrine mechanisms, it should be better clarified whether these effects are prominently attributable to endothelial-derived or also to cardiac-derived ANP (Figure 2). ANP increases systemic endothelial permeability through endothelial NPR-A, thereby maintaining intravascular volume homeostasis. Cardiac ANP reduces lung endothelial permeability in pathological conditions [110]. It should also be noted that most of the evidence regarding the endothelial effects of ANP were observed in vitro. In addition, most of the studies only investigated the contribution of exogenous ANP, without exploring the impact of endogenous endothelial-derived ANP.

## 6. Local Actions of BNP and CNP in the Cardiovascular System

Accumulating lines of evidence also suggest a local action of BNP and CNP in the cardiovascular system, although to a lesser extent than ANP. For example, in mice lacking BNP gene (BNP KO) cardiac fibrosis was observed 7 days after pressure overload. However, no signs of ventricular hypertrophy were evident in BNP KO mice [111]. Infusion of BNP was found to inhibit TGF-β-induced cardiac fibroblast proliferation and to reduce the expression of genes involved in fibrosis, myofibroblast conversion, and inflammation. These effects were mediated by the activation of PKG and mitogen-activated protein kinase (MEK)/extracellular signal-regulated kinase (ERK) pathway [112].

CNP was shown to be secreted by cardiac fibroblasts exposed to pro-fibrotic stimuli. CNP secretion by cardiac fibroblasts leads to the reduction of collagen synthesis in a cGMP dependent manner [32]. These findings were supported in vivo in rats subjected to myocardial infarction induced by coronary ligation. CNP administered for 2 weeks starting 4 days after myocardial infarction was found to decrease left ventricular enlargement, cross-sectional area of cardiomyocytes, and markers of cardiac fibrosis and hypertrophy, independently of changes in blood pressure [113]. Similarly, mice with cardiac overexpression of CNP undergoing myocardial infarction showed the decrease in cardiac hypertrophy and an amelioration of cardiac function after 3 weeks of permanent ligation of left coronary artery [114]. Consistently, rats overexpressing a dominant-negative form of NPR-B developed cardiac hypertrophy at 6 months of age, which was accelerated in animals undergoing chronic volume overload by infrarenal aortocaval shunt and studied 8 weeks after surgery. These data suggest the role of NPR-B in the regulation of hypertrophy [115]. In addition, Izumiya et al. found that chronic CNP administration (2 weeks) concomitantly with Ang-II treatment attenuated Ang-II-induced cardiac hypertrophy without affecting blood pressure in mice. In this case, the effects of CNP were associated with the decrease of ROS and NOX4 expression [116].

Other works suggest that endothelial-derived CNP acts as an important regulator of blood pressure. In fact, endothelial specific CNP KO mice (CNP-ecKO) develop hypertension, atherogenesis, aneurysm, and showed an impaired endothelial-dependent vasorelaxation [117,118]. Conversely, no effects on blood pressure levels and acetylcholine-induced vasorelaxation in isolated arteries were reported in vascular smooth muscle cell (VSMC)-specific NPR-B KO model, which also preserved. The results indicate that CNP is required for the maintenance of blood pressure and endothelial function independently of VSMC NPR-B. However, the effect of CNP in the induction of vasodilation was abolished in VSMC NPR-B KO [117]. Spiranek et al. further demonstrated that the action of CNP on vasodilatation is strictly dependent on vessel diameter. In fact, administration of CNP induced a vasodilatation on precapillary arterioles and capillaries and did not affect proximal arterioles. The vasodilatory effects of CNP was preserved in mice lacking NPR-B in endothelial cells, whereas it was abolished when NPR-B was deleted in microcirculatory VSMCs and in pericytes. In these models, a peripheral resistance and chronic arterial hypertension was observed, with a preserved renal function [119].

The involvement of NPR-C in mediating the vascular protection exerted by CNP was also investigated. For example, Moyes et al. reported that NPR-C agonists were able to induce vasorelaxation and to lower blood pressure in wild-type mice [118].

CNP also plays an important role in vascular remodeling. For example, it was previously reported that CNP induces vasodilatation of human forearm resistance vessels in an endothelium-independent manner [120]. Of note, CNP was also found to reduce VSMC proliferation induced by platelet-derived growth factor (PDGF) and to reduce intimal lesions in rat common carotid arteries after vascular injury [121,122]. Finally, CNP suppresses inflammation and fibrosis in rabbit carotid arteries subjected to shear stress, by the enhancement of NO production [123].

## 7. Therapeutic Interventions Targeting NPs

The evidence described here suggests that NPs act as critical regulators of cardiac and vascular homeostasis. As a consequence, the modulation of NPs may represent a useful strategy for the prevention or treatment of cardiovascular diseases. In this regard, intravenous administration of ANP was reported to improve cardiac remodeling in patients with AMI undergoing primary coronary angioplasty [98]. ANP was also proven to improve cardiac function in patients with AMI, without affecting blood pressure [99]. However, a renal resistance to ANP was reported in patients with chronic HF (CHF). In this regard, both in preclinical models and in patients, ANP treatment was found to attenuate diuresis and natriuresis [124,125,126,127,128,129]. Moreover, patients with congestive heart failure showed sodium retention and oedema despite high levels of circulating cardiac NPs [130]. Renal resistance to NPs, defined as the “endocrine heart paradox”, may be explained by three main mechanisms, as reviewed in detail by Clerico et al. [128]: (i) inactivation of circulating NPs, (ii) downregulation of NPRs, and (iii) by mechanisms acting downstream to NPRs.

Apart from ANP administration, synthetic NPs have been developed and tested in different clinical trials. Anaritide and carperitide are synthetic forms of ANP, whereas neseritide and cenderitide are the recombinant forms of BNP and CNP, respectively [131,132]. It was reported that neseritide improves hemodynamic function and global clinical status, such as dyspnea and fatigue in patients with congestive HF [132,133]. However, it is now known that safety and efficacious of neseritide appear to be low. A previous large clinical trial conducted in patients with acute HF showed that neseritide was not able to significantly reduce mortality and alleviate symptoms in these subjects [134]. In the same study, neseritide increased hypotension. In addition, two meta-analyses revealed that neseritide impairs renal function and increases the risk of short-term mortality in acutely decompensated HF patients [135,136]. These detrimental effects appear to be mediated also by hypotension induced by the drug.

ARNi, angiotensin receptor–neprilysin inhibitors, are able to enhance circulating levels of NPs, as previously reported. To date, ARNi have been shown to be efficacious in patients with HF with reduced ejection fraction (HF-REF) [61]. In the PARADIGM-HF trial, LCZ696 (sacubitril/valsartan), also known as ENTRESTO, was shown to reduce mortality and morbidity in patients with HF-REF in a manner more efficacious than enalapril alone [137,138]. However, the effects of LCZ696 on NEP inhibition in acute heart failure requires further investigations. In fact, Vodovar et al. reported that elevated levels of BNP (916 pg/mL) correlated with a decrease of NEP activity in patients with acute decompensated HF. BNP also inhibits NEP in vitro [139]. Thus, it is not clear the potential impact of high BNP levels on the efficacy of ENTRESTO in patients with acute HF [59].

The effects of ARNi are being testing also in ongoing clinical trials, in the setting of cardiac remodeling [140] and endothelial function (ClinicalTrials.gov Identifier: NCT03119623). Pre-clinical evidence about the use of ARNi in cardiac and vascular remodeling are promising. For example, LCZ696 used at a dose that does not lower blood pressure, was recently reported to reduce cardiac rupture and survival in a mouse model of myocardial infarction. The beneficial effect of LCZ696 was associated with the suppression of pro-inflammatory cytokines interleukin (IL)-1β and IL-6 and extracellular matrix degradation. The latter was achieved by the reduction of metalloprotease-9 (MMP-9) activity and expression [141]. In addition, sacubitril/valsartan was recently demonstrated to improve cardiac remodeling and to reduce infarct size in experimental models of myocardial infarction [142,143]. Besides ARNi, omapatrilat is an anti-hypertensive agent that combines neprylisin and angiotensin-converting enzyme (ACE) inhibition. Omapatrilat was shown to be an efficacious antihypertensive agent and to be a promising drug in a phase II and phase III HF trial [144,145]. However, it failed to reduce death and hospitalization in the OVERTURE clinical trial, in patients with CHF [146]. Adverse effects were also reported for omapatrilat. For example, it was shown that omapatrilat causes symptomatic hypotension and increases the risk of angioedema if compared to the administration of the only ACE inhibitor [145,146].

## 8. Conclusions and Perspectives

In this review, we summarized the relevant literature about the role of NPs, and in particular of ANP, in the improvement of cardiac and vascular remodeling in different stress conditions. In vivo and in vitro studies revealed that local ANP secretion by cardiac and endothelial cells play important pleiotropic functions through autocrine and paracrine effects. This evidence suggests that ANP acts as a main regulator of cardiovascular homeostasis in an autocrine and paracrine manner. Overall, the enhancement of ANP levels may be a promising target for the prevention and treatment of cardiovascular diseases, in both hypertensive and not hypertensive patients. In this regard, inhibition of NEP may be the appropriate strategy to potentiate the effects of NPs. However, some issues regarding pleiotropic effects of NPs in the cardiovascular system should be elucidated. First of all, the molecular mechanisms underlying the autocrine and paracrine functions of ANP need to be dissected. Secondly, downstream effectors of the cGMP/PKG pathway involved in the anti-remodeling actions of NPs should be identified. Thirdly, additional studies are needed to elucidate the effects of NPs in the vascular system, especially those related to metabolic alterations. Finally, it should be assessed in the future whether the modulation of NPs should contribute to the improvement of the clinical management of individuals at high risk of developing cardiac and vascular damage.
